# P11 The ratio and antibiotic resistance profiles of *Serratia* species among other causative bacteria isolated from blood cultures between 2015 and 2020

**DOI:** 10.1093/jacamr/dlac004.010

**Published:** 2022-02-16

**Authors:** Nida Özcan, Selahattin Atmaca, Erdal Özbek

**Affiliations:** Department of Medical Microbiology, Faculty of Medicine, Dicle University, Diyarbakır, Turkey; Department of Medical Microbiology, Faculty of Medicine, Dicle University, Diyarbakır, Turkey; Department of Medical Microbiology, Faculty of Medicine, Dicle University, Diyarbakır, Turkey

## Abstract

**Background:**

*Serratia* spp., especially *Serratia marcescens*, have become one of the main drug-resistant causes of hospital infections in the last five decades.^1^ There are a limited number of publications on *Serratia* spp., which cause sporadic infections or outbreaks in ICU patients, especially paediatric patients.^2^  *S. marcescens* was reported to have intrinsic resistance to many β-lactam antibiotics, tetracyclines and polymyxins.^3–5^

**Objectives:**

To investigate the antibiotic resistance profiles of the *Serratia* spp. and detection rates among blood cultures.

**Materials and methods:**

This retrospective study was approved by Dicle University Medicine Faculty Non-Invasive Clinical Research Committee (no: 361, 1 September 2021). Blood culture samples sent from Dicle University Hospital clinics and ICUs between 2015 and 2020 were included. Blood culture samples were incubated in the BD BACTEC FX (Becton Dickinson, USA) system, and the isolates were identified at genus and/or species level by MS using the MALDI Biotyper 3 (Bruker Daltonics, USA). Antimicrobial susceptibility tests (AST) of the isolates were performed with the BD Phoenix 100 (Becton Dickinson, USA) automated system. AST results were interpreted according to the EUCAST criteria.^6^

**Results:**

Among 9730 agents isolated from blood cultures over a 6 year period, 69 (0.7%) were identified as *Serratia* spp., 56 of them being *S. marcescens* (Table 1). Of patients from whom *Serratia* spp. were isolated, 37 (54%) were paediatric and 47 (68%) were ICU patients (Table 2). A total of 20 isolates (29%) were resistant to at least one of the carbapenems tested. The most effective antibiotics against *Serratia* spp. were found to be trimethoprim/sulfamethoxazole, ciprofloxacin and amikacin, with resistance rates of 3%, 4% and 7%, respectively (Table 3).

**Conclusions:**

Serratia species were isolated from blood cultures at a rate of 0.7% in a 6 year period, and increased carbapenem resistance among isolates was noteworthy.

**Table 1.** Species distribution of isolated *Serratia* spp.

*Serratia* spp.	*n* (%)
*S. marcescens*	56 (81.0)
*Serratia ureilytica*	3 (4.5)
*Serratia plymuthica*	1 (1.5)
*Serratia ficaria*	1 (1.5)
*Serratia rubidaea*	1 (1.5)
Unidentified species	7 (10.0)
Total	69 (100.0)

**Table 2.** Distribution of *Serratia* spp. grown in blood samples by patient (adult/paediatric) and services

Patients	Clinical services	*n* (%)
Paediatric patients (*n = *37)	ICU (*n = *28)	
	Paediatric ICU	21 (30.4)
	Neonatal ICU	6 (8.7)
	Burn unit	1 (1.4)
	Clinics (*n = *9)	
	Paediatric clinic	9 (13)
Adult patients (*n = *32)	ICU (*n = *19)	
	Internal medicine ICU	5 (7.2)
	Thoracic diseases ICU	4 (5.8)
	Neurology ICU	3 (4.4)
	Others	7 (10.2)
	Clinics (*n = *13)	
	Haematology/oncology department	6 (8.7)
	Others	7 (10.2)
Total		69 (100.0)

**Table 3.** Antibiotic resistance profiles of isolated *Serratia* spp. strains

Antibiotic	Resistant, *n* (%)	Intermediate, *n* (%)
Aztreonam	12 (17)	—
Ceftazidime	7 (10)	2 (3)
Ceftriaxone	12 (17)	1 (1)
Cefepime	10 (14)	1 (1)
TZP	17 (25)	—
SXT	2 (3)	—
Gentamicin	5 (8)	2 (3)
Amikacin	5 (7)	—
Ciprofloxacin	3 (4)	—
Imipenem	15 (22)	5 (7)
Meropenem	7 (10)	6 (9)
Ertapenem	20 (29)	—

TZP, piperacillin/tazobactam; SXT, trimethoprim/sulfamethoxazole.

## References

[1] Mahlen SD . *Serratia* infections: from military experiments to current practice. Clin Microbiol Rev 2011; 24: 755–791. 21976608 10.1128/CMR.00017-11 PMC3194826

[2] Cristina ML, Sartini M, Spagnolo AM. *Serratia marcescens* infections in neonatal intensive care units (NICUs). Int J Environ Res Public Health 2019; 16: 610. 10.3390/ijerph16040610 PMC6406414 30791509

[3] Liou BH, Duh RW, Lin YT *,* et al. A multicenter surveillance of antimicrobial resistance in *Serratia marcescens* in Taiwan. J Microbiol Immunol Infect 2014; 47: 387–393. 23751769 10.1016/j.jmii.2013.04.003 PMC7105062

[4] Stock I, Grueger T, Wiedemann B. Natural antibiotic susceptibility of strains of *Serratia marcescens* and the *S. liquefaciens* complex: *S. liquefaciens sensu stricto*, S. proteamaculans and S. grimesii. J Antimicrob Chemother 2003; 51: 865–885. 10.1016/s0924-8579(02)00163-2 12842326

[5] EUCAST. TEC on AST. Intrinsic Resistances and Unusual Phenotypes, Version 3.2. 2020. http://www.eucast.org/expert_rules_and_intrinsic_resistance.

[6] EUCAST. *Breakpoint Tables for Interpretation of MICs and Zone Diameters, Version 8.0*. 2018.

